# The safety and efficiency of retroperitoneal laparoscopic adrenalectomy via extra and intra perinephric fat approaches: a retrospective clinical study

**DOI:** 10.1186/s12893-019-0648-8

**Published:** 2019-12-21

**Authors:** Xuejian Wang, Junqiang Liu, Aozhang Ji, Changli Liu, Sony Nahayo, Lina Wang, Xinqing Zhu, Weiwei Fan, Xishuang Song, Jianbo Wang, Deyong Yang

**Affiliations:** Department of Urology, First Affiliated Hospital of Dalian Medical University, 116011, Zhongshan Road No, Dalian, 222 China

**Keywords:** Retroperitoneal laparoscopic adrenalectomy, Extra perinephric fat approach, Intra perinephric fat approach, Safety, Efficiency

## Abstract

**Background:**

This retrospective clinical study is to evaluate the safety and efficiency of two different approaches in retroperitoneal laparoscopic adrenalectomy and provide experience and basis for the treatment of adrenal tumors through retroperitoneal approach.

**Methods:**

From July 2015 to February 2018, 112 patients with adrenal lesions underwent retroperitoneal laparoscopic adrenalectomy (RLA) using a 3-port method. Among them, 56 patients underwent RLA via the extra perinephric fat approach (EPFA), 56 patients underwent RLA via the intra perinephric fat approach (IPFA). Clinical data, including preoperative, operative and postoperative management were recorded.

**Results:**

All surgeries were successfully completed, and there was no single patient who died during these surgeries. There was no statistically significant difference between the two groups in blood loss, postoperative complications, vena cava injury, renal cortex injury, peripheral organ injury, and post operation hospital stay. Peritoneum injury occurred more frequently in the EPFA group when compared with the IPEA group (*p* = 0.042). The average surgery time of the IPEA group is significantly shorter when compared with that of the EPEA group (*p* < 0.001). Due to serious saponification of the perinephric fat and heavy adhesion to renal fascia, three cases in IPFA group were converted to the EPFA surgery.

**Conclusion:**

RLA is a safe and effective procedure both via extra perinephric fat and intra perinephric fat approaches. IPEA is superior to EPEA in terms of peritoneal injury and duration. The choice may mainly depend on the experience of the surgeon, the characteristics of the adrenal tumor and the nature of the perinephric fat.

## Background

Laparoscopic adrenalectomy has become the golden standard approach for small benign adrenal tumors [1, 2]. This minimally invasive method shows obvious advantages over the traditional open surgery in perioperative complications and patient recovery [3].

Transperitoneal laparoscopic adrenalectomy is considered as a classic approach in adrenal surgery. However, since the retroperitoneal approach does not interfere with the abdominal organs, it has been proven to be a popular alternative for adrenal surgery [4–6]. In retroperitoneal approach, compared with the transperitoneal approach, the anatomic position of adrenal gland is concealed, the space of retroperitoneal cavity is relatively narrow and the anatomic marks are fewer and not obvious [7]. Thus, how to expose, separate and dissect the adrenal tumor through the optimal surgical route has become the key point of the laparoscopic operation of adrenal tumor through retroperitoneal cavity.

In this study, patients who underwent extra perinephric fat approach (EPFA) retroperitoneoscopic adrenalectomy were compared with those who underwent intra perinephric fat approach (IPFA). The anatomic landmarks and operative methods of each approach were described in detail, and the safety and efficiency of the two retroperitoneoscopic adrenalectomy approaches were evaluated and compared with each other. Finally, we explore how to select the optimal surgical approach according to the clinical characteristics of adrenal tumors so as to provide experience and basis for the treatment of adrenal tumors through retroperitoneal approach.

## Methods

### Patients

From July 2015 to February 2018, there were 112 patients in total who underwent retroperitoneoscopic adrenalectomy in our Institution. According to the difference of the surgical approach, they were divided into two groups. The number of patients per type of operation were classified as follows: 56 patients underwent laparoscopic retroperitoneal adrenalectomy through the extra perinephric fat approach, and 56 patients underwent laparoscopic retroperitoneal adrenalectomy through the intra perinephric fat approach.

A thorough and detailed history of all patients was provided preoperatively. Preoperative endocrinal investigations, including plasma cortisol, aldosterone, renin activity, catecholamine, adrenaline and noradrenaline, were routinely performed for all patients. Serum electrolyte levels were also evaluated. All the tumors were confirmed by CT or MRI. Patients’ variables were collected including age, sex, body mass index and presence of major comorbidities. Tumor characteristics including tumor size, location, and pathologic type were also recorded. Main surgical outcomes were collected including operative time, estimated blood loss, length of hospital stay, and complications.

### Surgical technique

Both the two approaches were performed by a single surgeon with one assistant, and all need three trocars (5 mm, 10 mm and 12 mm) for RPA. The patients were placed in lateral decubitus position with the affected side facing upward and the operative bed flexed just above the level of the iliac crest. The retroperitoneal space is entered posterior through a 20 mm transverse incision near to the tip of the 12th rib. A medial 10 mm trocar is placed along the border of the paraspinal muscle at a 45-degree angle pointing directly at the adrenal gland. A lateral 5 mm trocar is placed at the tip of the 11th rib. When the trocars were placed, the carbon dioxide gas was injected to maintain the retroperitoneal gas pressure at 20–25 mmHg. The first step was to clean the retroperitoneal fat tissue outside of Gerota’s fascia using a harmonic scalpel near the diaphragm until the fat tissue prolapses downward to the iliac fossa. The management of the central vein is a critical step in adrenalectomy, and improper management can cause massive hemorrhage. Preservation of the adrenal vein may not be compulsory [8, 9]. In this study, the preservation of the central adrenal vein depends on the actual condition of the tumor. In partial adrenalectomy cases, the tumors are small and far away from the central adrenal vein, the surgeon considers that preserving veins may be beneficial to patients. However, in total adrenalectomies cases which large tumors or highly suspected malignant adrenal tumors, it is imperative to ligate the vein [10]. Generally speaking, the central vein should be clipped close to the proximal end of the tumor in IPFA group while being clipped at the distal end in EPFA group. Whatever approach is adopted, the adipose tissue around the central vein should be cleaned up to the greatest extent, and the field of vision should be fully exposed to ensure safety. Then, the subsequent steps of the two approaches were different from each other.

### The extra perinephric fat approach (EPFA)

**Fig. 1:**
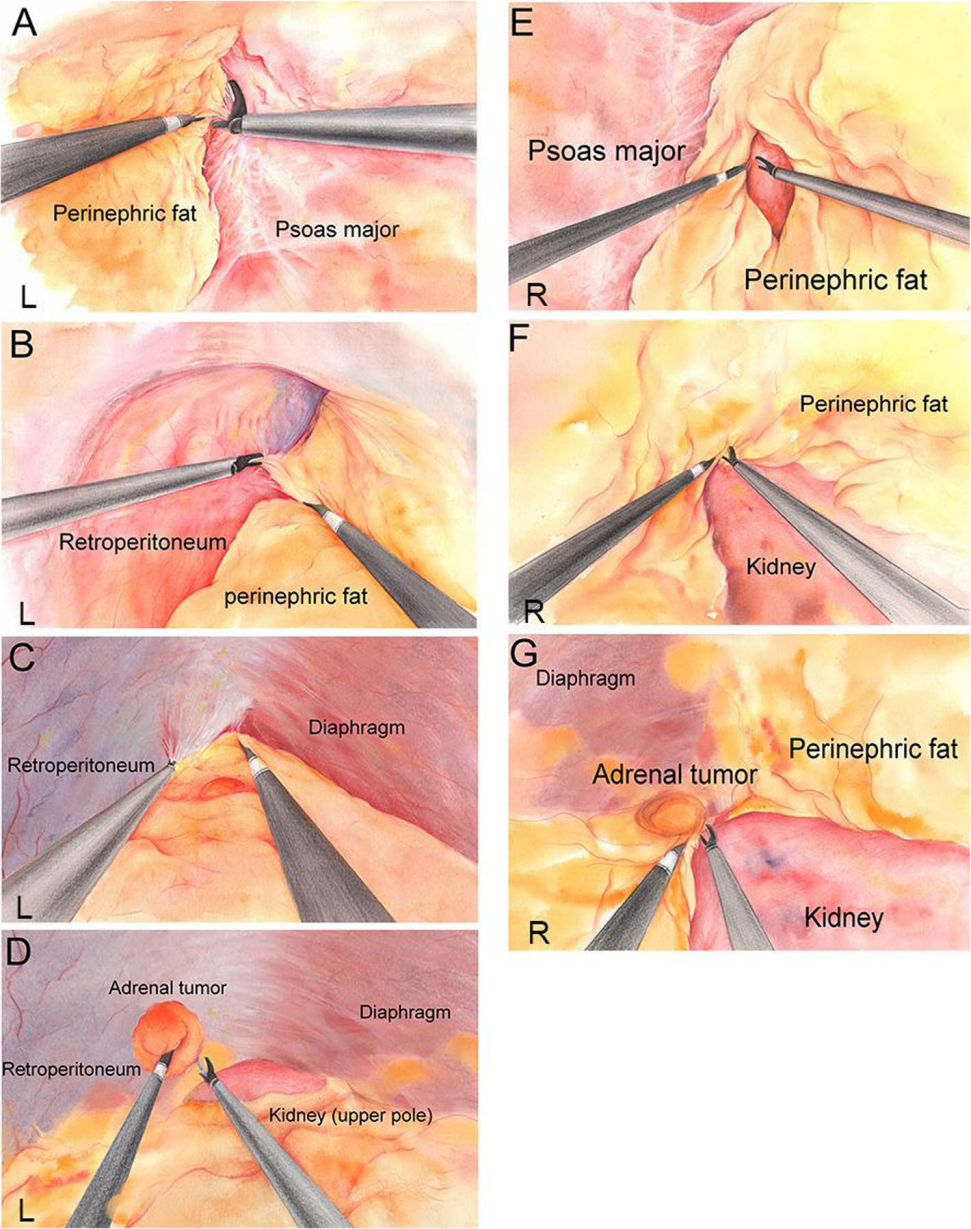
Surgical procedure of two different approaches A-D: Surgical procedure of EPFA. **a**: the first dissection plane is between the perinephric fat and psoas major muscle. **b**: The second dissection plane, turning to the ventral side, is between the perinephric fat and the retroperitoneum. **c**: The third dissection plane is between the diaphragm and the perinephric fat outside the adrenal gland and the upper pole of the kidney. **d**: Through the avascular plane between the adrenal gland and the upper pole of the kidney, the adrenal gland is dissected. **e-g**: Surgical procedure of IPFA. **e**: the perinephric fat capsule is opened longitudinally, **f**: Dissecting the interval between perinephric fat and the surface of the kidney on the ventral side. **g**: Looking for the adrenal gland above the upper pole of the kidney.

In EPFA, the first dissection plane is between the perinephric fat and psoas major muscle. The second dissection plane, turning to the ventral side, is between the perinephric fat and the retroperitoneum. At last, the third dissection plane is between the diaphragm and the perinephric fat outside the adrenal gland and the upper pole of the kidney. After the above three anatomic planes have been dissected, the retroperitoneal space becomes larger enough for the subsequent dissection of the adrenal gland. Through the avascular plane between the adrenal gland and the upper pole of the kidney, the adrenal gland is dissected. For the partial adrenalectomy, the central adrenal vein should be preserved while for the total adrenalectomy it may be clipped and transected. The arterial branches of the adrenal gland can be cauterized directly with the harmonic scalpel and then the adrenal gland is resected (Fig. 1. a-d).

### The intra perinephric fat approach (IPFA)

In IPFA, after incision of the Gerota’s fascia, the perinephric fat capsule is opened longitudinally, and then dissecting the interval between perinephric fat and the surface of the kidney on the ventral side. Keeping the perinephric fat attached to the retroperitoneum, and pushing the kidney downward so that the pressure from the pneumoperitoneum can help to create a wider operating space. The next step is to look for the adrenal gland above the upper pole of the kidney. In most cases, the adrenal gland is dimly visible above the upper pole of the kidney, however, in obese patients, further cleaning of the perinephric fat tissue around the adrenal gland is needed to locate the adrenal gland. Then, the procedure of the total or partial adrenalectomy is more or less the same to that in EPFA (Fig. 1. e-g).

### Statistical analysis

SPSS 21.0 software was used for statistical analysis. In case of normality, continuous outcomes are displayed as (means±standard deviation) and compared using the independent sample test. In case of a skewed distribution, measurement data use rank sum test. Counting data using chi square test. A *P*-value of < 0.05 was considered to be statistically significant for all tests.

## Results

Patients and tumor characteristics before surgery including age, sex, BMI, tumor location, mean tumor size, presence of major comorbidities and postoperative pathology results are illustrated in Table 1, and both groups were compared. All the tumors were removed completely and had sufficient specimens for a precise pathologic diagnosis.

**Table 1:** patient and tumor characteristics

	EPFA (*n* = 56)	IPFA (n = 56)	P
Age (years)	55.44 ± 12.07	51.26 ± 12.13	0.070
Sex			
male	25	34	0.013
female	31	22	
BMI (kg/m^2^)	25.83 ± 4.83	27.35 ± 3.66	0.226
Sides			
Right	28	24	0.527
Left	28	32	
Heypertension	36	42	0.304
Diabetes	12	11	0.815
Diameter (cm)	3.04 ± 1.30	2.60 ± 1.24	0.073
Diagnosis			
Nonfunctioning tumor	44	33	
Pheochromocytoma	4	1	
Aldosteronoma	7	21	
Cushing syndrome	1	0	
metastatic carcinoma	0	1	
Pathology			
Cortical adenoma	43	47	
Neurilemmoma	2	1	
Pheochromocytoma	5	2	
Cortical nodular hyperplasia	2	2	
Ganglion cell neuroma	1	0	
Angiomyolipoma	1	1	
Hemangioma	1	0	
Myelolipoma	1	1	
Poorly differentiated carcinoma (metastatic carcinoma)	0	1	

*EPFA* extra perinephric fat approach, IPFA: intra perinephric fat approach

BMI: Body Mass Index

The intraoperative and postoperative characteristics are shown in Table 2. All surgeries were successfully completed, and there was no single patient who died during the operations. There was no statistically significant difference between the two groups in blood loss, postoperative complications, vena cava injury, renal cortex injury, peripheral organ injury, and post operation hospital stay.

**Table 2:** Intraoperative and postoperative characteristics

	EPFA *n* = 56	IPFA *n* = 56	P
Mortality	0	0	
Mean operative time (min)	81.79 ± 27.54	63.63 ± 20.66	< 0.001
Conversions	0	3	0.079
Intraoperative complications			
Blood loss^a^ (HB)	22.7 ± 13.6	21.0 ± 11.9	0.492
Peritoneum injury	4	0	0.042
Vena cava injury	2	1	0.558
Renal cortex injury	0	2	0.135
Peripheral organ injury^b^	0	0	
Postoperative complications	0	0	
Postoperation hospital stay (days)	4.62 ± 2.99	5.44 ± 1.54	0.071

EPFA: extra perinephric fat approach, IPFA: intra perinephric fat approach

Conversions: continue the operation from IPFA to EPFA;

a:The differences of hemoglobin before and the third day after operation

b: Including the intestines, the pancreas, the liver, the spleen

Peritoneum injury occurred more frequently in the EPFA group when compared with the IPEA group (*p* = 0.042). There were two renal cortex injury which occurred in the IPEA group, and no renal cortex injury occurred in the EPFA group, however, when compared with each other, there was no obviously statistically significance (*p* = 0.135). The average operative time of the IPEA group is significantly shorter when compared with that of the EPEA group (*p* < 0.001). Due to serious saponification of the perinephric fat and heavy adhesion to renal fascia, three cases in IPFA group were converted to EPFA surgery.

## Discussion

Laparoscopic adrenalectomy was first described in 1992 by Gagner et al. [11] and, since then, has progressively replaced conventional open adrenalectomy as the gold standard procedure for the treatment of benign adrenal disease. Compared to open adrenalectomy, laparoscopic adrenalectomy has proved to be advantageous in lower blood loss, shorter hospital stay, and lower complication rates [3]. Recently, robotic platform plays a leading role in urology surgery. However, in terms of adrenalectomy, robot-assisted adrenalectomy may seem to be luxurious and its adoption may be done only in selected patients for whom laparoscopic adrenalectomy is extremely challenging [12]. Thus, for the cost-effectiveness consideration, it is generally acknowledged that laparoscopic surgery is still the mainstream in adrenalectomy.

Retroperitoneal approach offers direct access to the adrenal gland without the need for visceral mobilization or lysis of adhesions from prior abdominal operations [6, 13]. In these retroperitoneoscopic adrenalectomy series, both the extra perinephric fat approach and the intra perinephric fat approach can be used effectively. There was no difference in patient position and trocar configuration between the two approaches. The 12 mm trocar was kept on the right side of the operator, for the right-handed surgeon, to make it easy to operate when complex operations are encountered. No serious complications occurred during the surgery of all patients included. There were no significant differences between the two groups in terms of intra- and post-operative implication rate as well as duration of the hospital stay. The only obvious difference between the two groups is that the operation time is shorter in the intra perinephric fat approach group when compared with that of the extra perinephric fat approach group.

In the extra perinephric fat approach of retroperitoneoscopic adrenalectomy, the larger operative field of this approach helps with a better orientation and visualization of familiar anatomic landmarks [7], which is particularly helpful during the early learning curve. A larger working space is useful for the removal of larger adrenal masses. The anatomical landmarks in this approach include psoas major, peritoneal reflexion and diaphragm. The risk of kidney injury is low due to the fact that the tissue is completely dissected outside of the perinephric fat. However, in patients with thinner retroperitoneum, the risk of retroperitoneal injury is higher when dissecting the ventral plane. Even more, if the perinephric fascia adheres to the retroperitoneum, care should be taken during dissecting so as not to cause retroperitoneal injury, or even injury to organs such as the pancreas and intestines. If carefully separated, the incidence of retroperitoneal injury should be extremely low. In this cohort study, there were 4 cases with retroperitoneal injury, all of which were minor ruptures. Retrospective video showed that 1 case was caused by sudden separation and 3 cases were caused by adhesion of retroperitoneum to Gerota’s fascia. However, peritoneal injury can be easily repaired by clipping or suturing under laparoscope. Careful intraoperative separation and familiarity with anatomical layers are the keys to avoid injury.

In the extra perinephric fat approach, when the diaphragmatic plane is dissected, the adrenal gland loses its suspension and moves down with the kidney, which makes it difficult to remove the adrenal tumor in the next step. Thus, in some cases, we modify the procedure of the extra perinephric fat approach as the following, when the dorsal and ventral planes are separated, separation along the adrenal gland from the upper pole of the kidney keeps the adrenal gland suspended on the diaphragmatic plane, which is beneficial to the next step of adrenalectomy. The main advantages and disadvantages of the two different approaches are shown in Table 3.

**Table 3:** Main advantages and disadvantages of the two different approaches

EPFA	IPFA
Larger operative field, familiar anatomic landmarks	Narrow space, lack obvious anatomic landmarks
Helpful during the early learning curve	Not suitable for beginners
Less affected by the nature of perinephric fat	Greatly affected by the nature of perinephric fat
Prone to retroperitoneal injury	Prone to renal fascia laceration, renal arenchyma injury
More dissection, longer operative time	Less dissection, shorter operative time

In general, the adrenal gland is located next to the upper pole of the kidney. This anatomic neighborhood location relationship provides a basis for intra perinephric fat approach of retroperitoneoscopic adrenalectomy. This surgical approach requires a smaller range of tissue dissection and is able to reach the adrenal gland more directly and quickly, thus the reduction of the surgery time. When the adrenal gland was found, it is suspended on the diaphragm by the original fat structure, and the position is relatively fixed and this is helpful for the subsequent dissection of the vessels and the masses. Since the tissue is dissected within the perinephric fat, the risk of retroperitoneum injury, abdominal organs such as pancreas, intestines, and spleen injury is low.

The space in the intra perinephric fat approach is relatively narrow and lacks obvious anatomic marks. The main anatomic marker is the upper pole of the kidney. Especially, for obese patients with thick perinephric fat, it may be difficult to find adrenal tumors. Therefore, it is not suitable for beginners to choose this surgical approach. While deciding to use the intra perinephric fat approach, it is necessary to assess the degree of perinephric fat adhesion according to the Mayo score [14]. If the perinephric fat adhesion or stiffness is serious, intraoperative complications such as a rupture of renal fascia and an injury of the kidney parenchyma may occur. In IPFA group, the position of opening perirenal fat was located at the level of middle lateral border of kidney. In determining the division plane of perirenal fat, some anatomical markers should be consulted. In the posterior abdominal cavity, the position of the arcuate ligament is at the level of the renal pedicle. For patients with thicker perirenal fat, an arcuate ligament can be chosen as an anatomical landmark, thereby, effectively determining the position of the kidney and improving the efficiency and safety of separating the perirenal fat. In addition, another problem to consider while using the intra perinephric fat approach is when the patient develops later a kidney tumor or other kidney disease that requires surgery, the procedure will be extremely difficult for the next operation.

For the surgeon, it is very important to choose the appropriate surgical approach. According to this study, the feature of peripheral kidney fat is the main factor to be considered when doctors decide on the kind of approach which might be more suitable for patients. While dissecting peripheral fat to the kidney, stiff or adherent peripheral fat may cause the procedure to be more complicated. Based on the Mayo Adhesive Probability (MAP), the main risk factors for AFP include posterior and lateral peripheral fat thickness and peripheral fat stranding on CT imaging. Thus preoperative evaluation of the AFP is crucial for deciding on the superior surgical approach. We prefer the EPFA approach for patients who were considered to have a stiffness or adherent PF. At the same time, some patients have adhesions of perirenal fat, especially in male patients the fatty tissue is often strongly attached to the kidney. This kind of adhesions and rigid adipose tissue will increase the complexity of IPFA. While dissecting perirenal fat, the instrument should be closely attached to the perirenal fascia for separation. If the renal fascia is easy to rupture and bleed, in order to avoid further injury, it is better to switch to EPFA.

In addition, we choose a different approach due to the different position of the gland in relation to the kidney, Generally speaking, the location of the right adrenal gland is higher than that of the left adrenal gland. For patients with lower tumor location, no matter which surgical approach is adopted, the kidney should be dissected with a wider range to reveal the surgical field of vision and ensure the safe and effective operation. In general, adrenal tumors whose size is less than four centimeters are mostly benign tumors, but the possibility of metastatic cancer or primary adrenal malignancy cannot be completely ruled out. If the preoperative diagnosis is highly suspected that the tumor is malignant, en-bloc resection of tumor and the peripheral fatty tissue is the reasonable choice, thus, it is probably oncologically more correct to choose EPFA. Appropriate surgical approach will bring the greatest benefit to patients.

## Conclusion

Retroperitoneoscopic adrenalectomy is a safe and effective procedure both via extra perinephric fat and intra perinephric fat approaches. IPEA is superior to EPEA in terms of peritoneal injury and duration. The choice may mainly depend on the experience of the surgeon, the characteristics of the adrenal tumor and the nature of the perinephric fat.

## Data Availability

The datasets used and/or analysed during the current study are available from the corresponding author on reasonable request.

## Abbreviations

EPFA: Extra perinephric fat approach
IPFA: Intra perinephric fat approach
MAP: Mayo Adhesive Probability
RLA: Retroperitoneal laparoscopic adrenalectomy
